# Protocol for using mixed methods and process improvement methodologies to explore primary care receptionist work

**DOI:** 10.1136/bmjopen-2016-013240

**Published:** 2016-11-16

**Authors:** Ian Litchfield, Nicola Gale, Michael Burrows, Sheila Greenfield

**Affiliations:** 1 Institute of Applied Health Research, College of Medical and Dental Sciences, University of Birmingham, Birmingham, UK; 2 School of Social Policy, University of Birmingham, Birmingham, UK

**Keywords:** PRIMARY CARE, HEALTH SERVICES ADMINISTRATION & MANAGEMENT

## Abstract

**Introduction:**

The need to cope with an increasingly ageing and multimorbid population has seen a shift towards preventive health and effective management of chronic disease. This places general practice at the forefront of health service provision with an increased demand that impacts on all members of the practice team. As these pressures grow, systems become more complex and tasks delegated across a broader range of staff groups. These include receptionists who play an essential role in the successful functioning of the surgery and are a major influence on patient satisfaction. However, they do so without formal recognition of the clinical implications of their work or with any requirements for training and qualifications.

**Methods and analysis:**

Our work consists of three phases. The first will survey receptionists using the validated Work Design Questionnaire to help us understand more precisely the parameters of their role; the second involves the use of iterative focus groups to help define the systems and processes within which they work. The third and final phase will produce recommendations to increase the efficiency and safety of the key practice processes involving receptionists and identify the areas and where receptionists require targeted support. In doing so, we aim to increase job satisfaction of receptionists, improve practice efficiency and produce better outcomes for patients.

**Ethics and dissemination:**

Our work will be disseminated using conferences, workshops, trade journals, electronic media and through a series of publications in the peer reviewed literature. At the very least, our work will serve to prompt discussion on the clinical role of receptionists and assess the advantages of using value streams in conjunction with related tools for process improvement.

**Strengths and limitations of this study**

- First study of its type to undertake an assessment of the parameters of receptionist work using the validated Work Design Questionnaire.
- We will gain an understanding of the tasks completed, the knowledge needed, the social support received and the context of their work.
- This will be the first work to have constructed value stream maps (VSMs) and service blueprints that identify areas of weakness and strength in the clinical processes in which receptionists are involved.
- We will make recommendations that aim to improve processes and directly support receptionists.
- The integration of rigorous research with state of the art tools of service improvement will itself draw attention to the findings and contribute to the methodology of improvement techniques.

## Introduction

The pressure on primary care in the UK is growing, consultation rates are on the increase and the workload on general practitioners (GPs) is mounting.^1^ This increased demand impacts on all members of the practice team as time pressures grow, systems become more complex and tasks are increasingly likely to be delegated across a broader range of staff groups.^2^ These include receptionists who play an essential role in the successful functioning of the surgery and are a major influence on patient satisfaction.^3^

As well as undertaking administrative and clerical duties to ensure the various office systems continue to support the delivery of care, such as filing, maintaining medical records and making appointments,^4^ ^5^ they also undertake functions more directly related to patient health, in particular booking appointments, communicating test results and managing repeat prescriptions. These responsibilities are placed on staff that are not required to undertake any related training, from data protection and information governance to styles of communication.^6^ The gap between training and the implication of the role has clinical consequences for patients and medicolegal concerns for practices where legal responsibility for errors involving receptionists is vague and where previous litigation has led to an assessment of how that task was designated and the competency of the receptionist involved.^7^ ^8^

Previous work has described how in satisfying these various functions, receptionists experience competing pressures from patients and GPs and feel isolated fulfilling a role with clear responsibility for patient health, often without appropriate support.^6^ ^9^ ^10^ In attempting to gain a greater understanding of the role of receptionists, previous research has focussed on their position at the practice front desk and the extent to which they are understood and valued by patients.^10^ ^11^ Fewer studies have examined the relationship with other members of the practice team and how they interact.^5^

In Australia, guidance for supporting receptionists has begun to emerge,^12^ ^13^ yet currently there is no UK national guidance for the key functions of receptionists, and existing training requirements are minimal.^6^ The attitude of receptionists toward their current role has not been fully explored and systematic consultation with all stakeholders to develop and implement policies and processes to support receptionists is absent. However, the increasing pressure on primary care resources indicates a need to improve the efficiency of the processes they are involved in and for this a more thorough understanding of the parameters of their role and experiences is required as well as an understanding of the site and nature of their interaction with the other elements of primary care delivery including staff, patients, materials and information.^14^ One tool frequently used by lean methodologies to identify these elements is the value stream map (VSM).^15^ This is a graphic representation of a set of activities and values involved in creating a product or providing a service previously used in manufacturing.^16–19^ These maps can be used to inform and complement service blueprints, a related tool originally used in the service industry to diagnose problems with operational inefficiency and highlight areas of potential error, delay and failure.^20^ ^21^

Here we describe a multiphase study that aims to help receptionists deliver robust, consistent and safe care, responsive to the needs of their employers and patients. To do this, we will first define the parameters of the roles and responsibilities of receptionists, use iterative discussions with receptionists, clinical and non-clinical general practice staff and patients to create VSMs and service blueprints^21^ to understand and contextualise the various roles and functions they perform. Then we will target our recommendations for increasing the efficiency of the support they might need and in what form.

### Knowledge review

Here we summarise the findings of our scoping review^22^ that describes existing knowledge of the key areas of receptionist work that possess direct clinical implications for patients. From this review, we identified areas which included managing appointments, reporting test results and repeat prescriptions. In addition, we looked at the discourse styles typically used by receptionists in dealing with patients and their implications for efficiency and patient satisfaction.

#### Managing appointments

Appointment making is a key role in general practice and can impact on patient satisfaction and outcomes.^23^ ^24^ While a contentious concept, in prioritising allocation of appointments non-medically trained staff are regularly making ‘triage’ decisions in general practice which can affect patient outcome.^7^ ^25–27^ Poor experiences of appointment making/contact with the practice can lead to costly or dangerous health outcomes including the patient visiting A&E.^28^ ^29^

Primary care organisations are ‘professional bureaucracies’ and administrative staff perform a key role in creating the boundary of the organisation, are able to exercise considerable discretion and so gain indirect and subtle power and able to exercise considerable discretion.^30–32^ This may go some way as to explaining why receptionists are often presented as powerful characters that make important judgements in uncertain conditions.^33–35^ However, booking appointments is a complex social process, often dependent on negotiation and factors such as patients' expectations and appointment availability.^9^ Reconciling demands and expectations of patients with availability of healthcare providers can expose them to social friction.^10^ There is a pay-off between access and continuity of care.^36^ Continuity is getting hard to achieve as demand increases and practice size and staff number do the same.^37^ In most cases, the process is not formalised and can be difficult to document, define and assess.^7^ Receptionists are exposed to social pressure from anxious patients and patients vulnerable to receptionists making potentially key decisions without the necessary and appropriate support. This may go some way to explain the considerable variability between general practices as to how the appointment making process is perceived by patients.^38^

In trying to improve consistency in booking appointments, previous research has indicated how appropriate guidelines can positively impact on negotiations of urgency and receptionists' relationships with patients and make it easier to prioritise patient appointments. Appealing to defined rules in negotiations with patients can be a useful source of legitimacy and support for receptionists.^10^ In Australia, standards have been produced that offer such guidance^39^ and there are recommendations for the roles and responsibilities for all staff managing patient appointments.^13^ It has been recommended that practices in the UK should also be more explicit in how they book appointments,^9^ and establish boundaries for reception staff in responding to telephone requests.^12^

#### Reporting results

In a recent UK survey of result communication in primary care, 98% reported that the default option of communicating normal results was for patients to call reception staff. A further 18% of practices required receptionists call patients with abnormal results.^40^ Feedback on result data should include information on the implications of the result, options for further care and emotional support offered.^41^ Yet receptionists are not required to undertake any training to fulfil this role and lack clinical expertise. Patients have previously expressed dissatisfaction with the level of information they receive on their laboratory test results.^42^ ^43^ The ensuing uncertainty about the meaning, or accuracy, of normal results can lead to additional costly and unnecessary medical visits and diagnostic procedures.^44–47^ If, however, receptionists were equipped to communicate more detailed and consistent information it may help reassure patients and encourage positive health behaviours.^48–51^

#### Repeat prescriptions

Repeat prescriptions are defined as those issued without a consultation between clinician and patient.^52^ The process of repeat prescribing is typically a complex, technology-supported social practice requiring the input of clinical and administrative staff.^53^ In the UK, repeat prescriptions account for three quarters of all drugs prescribed with half of all patients receiving treatment via repeat prescriptions.^52^ ^54–56^

Repeat prescribing has been recognised as a core element of the receptionist role,^11^ ^57^ one where they make extensive use of tacit knowledge and situated judgements to bridge the gap between the formal organisational routine and the actual routine as it plays out in practice.^58^ They make important hidden contributions to quality and safety in repeat prescribing and there is evidence they judge themselves accountable to patients for those contributions.^53^ Yet 4.9% of repeat prescription contain an error^59^ and considering the volume ordered this can have considerable impact on patients and resource.

#### Front of house communication

In all of the above, the receptionist is required to interact with patients. The receptionist is the key buffer between practice and patients and a recent survey of complaints in primary care found those concerning receptionists continued to grow and in 2014/2015 administrative staff were responsible for some 43% of upheld complaints, the largest number of any staff group.^60^ Patients can assume that receptionists find their enquiries disruptive and report feeling intimidated.^32^ ^61^ ^62^ Patients have cited their poor relationship with practice staff and receptionists as a reason for non-attendance.^63^ ^64^ This can be attributed to the ‘task-centred’ style of discourse receptionists frequently employ which can be perceived as overly direct, paying little attention to the voice of the patient ref. 11 pp. 571–7 and also seen as being less effective at meeting patients' needs than those with more patient-centred orientations.

Receptionists rely on objective information where available and subjective interpretations to judge the way that they interact with patients. Previous research has found that receptionists can undertake a ‘moral’ judgement on patients founded on a variety of factors including appearance, accent and ethnicity^65^ ^66^ and these can influence decisions about their suitability or acceptability for treatment and the access granted.^34^ ^67^

In trying to improve this interaction, evidence is beginning to emerge that suggests receptionists' communication is more effective and better received when patients are clear as to where the conversation is heading.^68^

### Using process improvement tools

#### Value added maps

In the UK and elsewhere, healthcare providers are increasingly relying on process improvement methodologies such as lean or six sigma, first used in the manufacturing industry to streamline production, increase efficiency and minimise waste.^16–19^ These methodologies require that existing systems of service provision are thoroughly understood.^14^ One key tool used to achieve this is the VSM. First used in manufacturing by Rother and Shook^69^ they comprise material and information flows necessary to transform a raw material into a final product; analogous in healthcare to transforming an unhealthy patient into a healthy one.^70^ These maps created in conjunction with multidisciplinary teams help identify which inputs and processes have the greatest impact on the desired output and so allow team members to design action plans, and generate and implement revised solutions.^71^

Many of the VSMs used in healthcare relate either to patient flow^72–74^ or information streams.^75^ ^76^ They are not designed to show both at the same time meaning exploring the interaction between various elements that combine to provide a service is problematic.^77^ We are therefore proposing that we use value maps in conjunction with service blueprints. These are a related service improvement tool that can grant an understanding of how ‘visible’ elements of the receptionists' work, for example, the communication of results from receptionists to patients can combine with ‘backstage’ elements, that is, the process that leads to the information on the result reaching the receptionist.^21^

### Summary

Within UK general practice, a number of administrative and clinical roles are fulfilled by the receptionist. In the process of fulfilling these critical functions they often bear the brunt of patient frustration, anxious for timely appointments, results or prescriptions. Guidance for receptionists as they undertake these activities is lacking as is an understanding of how we can streamline these processes to make them more efficient. We will therefore work closely with receptionists, practice staff and patients to understand the role of receptionists, offer them appropriate support and make recommendations for improving the key processes of which they are part.

## Methods and analysis

Our work consists of three key phrases that will, first, help us understand the parameters of the role of receptionists, second, the systems and processes they work within, third, identify areas of support for receptionists and recommendations with the potential to increase the efficiency. In doing so, we aim to increase job satisfaction of receptionists, improve practice efficiency and produce better outcomes for patients. We will work closely with receptionists, other practice staff and patients to produce recommendations for improving extant practice systems and produce guidance specifically for receptionists to support their clinical roles. Receptionists will have the opportunity to provide valued feedback about their current role, the design of improved practice systems and how more harmonious interactions with patients might be realised.

### Research questions

The study aims to answer two main research questions; first, can using work design questionnaires (WDQs), VSMs and service blueprints provide a greater understanding of the processes and influences on receptionists in their clinically relevant roles? Second, how can these questionnaires, maps and blueprints be used to inform recommendations for measurable process improvement and appropriate support for receptionists?

### Research design

We will conduct our work in three phases using a standard mixed-methods approach:^78^

*Phase I*: Establish the parameters of the current role of receptionists.

To do this we will use the validated WDQ to measure job and work characteristics of receptionists.^79^ The questionnaire has been validated by 540 incumbents holding 243 distinct jobs and has demonstrated excellent reliability and convergent and discriminant validity.^79^ The focus of the questionnaire is work design (as opposed to the narrower term job design) and it acknowledges the job and the link between this and the broader environment.^80^ The questionnaire seeks information on four key characteristics of the job. The first is task characteristics which concerns how the task is accomplished, and the range and nature of tasks of a particular job. Factors explored include autonomy, and the significance and variety each task entails. The second is knowledge characteristics reflecting the kinds of knowledge, skill and ability demands placed on an individual as a function of what is done on the job. This includes factors such as complexity, information processing and problem solving and the training provided. The third is social characteristics which relate to social support, interdependence, and external interaction with individuals not belonging to the organisation. The fourth and final set is contextual characteristics which look at elements of the interaction with the individual's environment including ergonomics, physical demands, work conditions and the equipment used including familiarity with electronic clinical support systems.

As part of this process, we will also gather data on receptionists' age, ethnicity, gender, and other personal characteristics protected by UK law as well as their years in post, and characteristics of the practice they work. The latter will include the number of GPs, patients and the identity of their commissioning group. The information we gather will provide the most detailed exploration of the characteristics of receptionists' work yet conducted in the UK and inform the topic guides to be used in Phase II. The output of these focus groups will help us evaluate the applicability of such WDQs in similar studies in the future.

*Phase II*: Creation of VSMs and Service Blueprints.

Using the output of focus groups with receptionists and other stakeholders (eg, patients, practice managers and GPs), we will create VSMs and service blueprints to determine practice systems and processes. This will allow us to make recommendations as to how practices might reduce delay and increase efficiency as well as identify which aspects of the role of receptionists require increased support.

#### Focus groups

We will use focus groups of between six and eight participants^81^ to explore the issues that emerge from the WDQ and in particular the role of receptionists in the three key tasks of communicating results, booking appointments and providing repeat prescriptions. Focus groups will be audio recorded and outputs, such as maps or graphical representation, from participants retained by the research group. The focus groups will consist singly of receptionists, a range of other practice staff and patients. We will retain the flexibility to carry out additional focus groups until saturation is reached. We will employ a team-based approach to analysing the discussions and use them to inform the VSMs and service blueprint.^82^ We will evaluate the validity of the VSMs and blueprints by presenting iterative drafts of both to subsequent focus groups.

#### Value stream maps

The maps will graphically represent each task as a series of steps using various shapes, symbols and colours to provide information on the type of action, the individual involved and any associated values. For clarity, we will populate the maps with a series of conventional symbols used in process maps introduced and refined by Gilbreth and Graham^83–85^ and follow the recommendations for using specific colours and icons to denote the identity of the various care providers.^14^

Where possible we will capture metrics such as cycle times, defect rates and wait times. Each map will provide the opportunity to understand the roles of various individuals, and the flow of materials and information required to support the receptionist's role.^18^ ^86^ ^87^ A systematic analysis of these maps will then help us identify areas that are wasteful or otherwise fail to provide ‘value’ to provide evidence of how work processes may be streamlined, reducing costs and increasing quality.^88^ ^89^

We are unsure as to how similar or different these processes may be across practices. If similar, then our intention is to produce maps that reflect the key elements of these and recommendations that once evaluated are transferable across sites. If the processes are markedly different between practices, then we will produce bespoke maps for each.

#### Service blueprints

Service blueprints clarify the interactions between service users, and service employees, including digital contact, the front-of-house activities that involve direct contact with patients, and the backstage activities that the customer does not see, that is, the processes and systems that underpin the delivery of each aspect of the service. They will be used to contextualise the corresponding viewpoints of practice staff, patients and external groups for the various receptionist workstreams identified in Phases I and II.^82^ ^90^

To ensure the maps and service blueprints serve the purpose of guiding process improvement, they will be analysed as consistently and systematically as possible by the members of the study team and objective decisions made as to any unnecessary steps, duplications/redundancies, variability, bottlenecks, delays and role ambiguity.^91^

*Phase III*: Recommendations for process improvement and support for receptionists.

We will use those areas identified in Phase II where current processes are either failing or introducing unnecessary delay to produce a series of recommendations to promote reshaping of current work processes. In addition, we will identify and recommend appropriate support for administrative staff. Taken together this will allow receptionists to offer a more efficient, robust and consistent service for patients.

### Settings and participants

Given the cultural variation that exists across UK practices as independent businesses,^58^ it is important to understand how these contextual differences impact on the work of receptionists.

*Phase I*: Primary care practices across England.

The WDQ will be made available online to receptionists at practices across England. To ensure sufficient power we will collect a minimum of 500 questionnaires. We will use survey software^92^ to manage the collection and collation of data.

*Phase II*: Primary care practices from the West Midlands.

We will conduct a series of focus groups at a minimum of four practices across the West Midlands to reflect maximum variance in size and location of practice including rural and urban settings and a variety of deprivation scores.^93^ At each of the four practice sites in the West Midlands, we will conduct a minimum of three focus groups consisting singly of receptionists, other practice staff and patients. All staff are eligible to participate with no restriction, except consent. Participants in patient groups will be drawn from the same practice to gain their perspectives on the role of receptionists, again with no restriction except ability and willingness to consent.

### Recruitment

*Phase I*: We will promote the study and the need for receptionists to complete the questionnaire using a mailshot and articles in generic trade journals, through the various Clinical Commissioning Groups (CCGs) and national primary care bodies such as the Royal College of General Practitioners (RCGP) as well as the Association of Medical Secretaries, Practice Managers, Administrators and Receptionists (AMSPAR) and The British Society of Medical Secretaries and Administrators (BSMSA). There are a number of ways of facilitating a questionnaire based survey each with their own benefits and limitations. Though self-selecting bias can play a role in postal surveys,^94^ self-administration of questionnaires can increase respondents' willingness to disclose sensitive information, compared with face-to-face or telephone interviews.^95–97^

*Phase II*: We will use the local Primary Care Research Network (PCRN) to identify suitable practices; these will be visited in person by a member of the study team and the broader aims of the study and the role and implications of involvement of the individual practices will be discussed with the practice staff. Patients will be recruited through existing patient groups at each practice and via posters in the practice and where possible other means of communication such as text messages from the practice to patients or mail-outs.

### Data management and analysis

#### Data management

Data collected from the focus groups will consist of an audio recording. These will be downloaded to and stored on an encrypted flash drive prior to leaving the data collection site. Following this, the recording will be transcribed either by a member of the research team or by a reputable transcription service. Data storage will be kept secure as per data protection guidelines.^98^ Hard copies of data will be stored in a secure and locked location and digital/electronic files will be securely stored and encrypted, with passwords. All data will also be backed-up; these too will also be stored securely. Other data collected may include maps created by the participants; these will be stored in accordance with the description of stored hard copies of data given.

#### Analysis of focus groups

We will analyse the focus groups in two ways; first, we will use a conventional framework based approach to analyse the focus group data.^99^ The data will be sifted, charted and sorted in accordance with key issues and themes. Framework analysis is typically used for applied or policy relevant qualitative research based on relatively structured data generation based on preset aims.^99^ Second, we will use the data from the focus groups to create VSMs of the three key clinically related processes outlined above.

#### Analysis of value stream maps

We will use group based deductive analysis of the VSMs to produce service blueprints and otherwise determine areas of strengths and weakness and highlight areas in the process where either delay or failure can be introduced. These will be used to inform our recommendations for improving current processes.

### Study outcomes

There are a number of key study outcomes related to each of the three phases. First, we will gain a greater understanding of the role of receptionists including the key parameters of the job as described by the results from the WDQ. Second, the VSMs and service blueprints will allow us to make recommendations to improve the three clinically related processes that receptionists contribute to. They will also allow us to target the areas where receptionists need support. In particular, we will make recommendations for the development of structured guidance for prioritising the booking of appointments, the management of repeat prescriptions and the content of result communication. As a result of these recommendations, we will raise awareness of patient confidentiality and improve information governance by receptionists. At an organisation level our work will increase awareness of the role of receptionists as a key member of the primary care team, it will increase efficiency and reduce the number of errors.

## Discussion

A key strategy of future healthcare is preventive health and effective management of chronic disease placing general practice at the forefront of health service provision in the UK and abroad. To meet this need, traditional models of primary healthcare delivery are changing with greater responsibility assumed by a broader range of practice staff. Long seen as fulfilling an important yet predominantly administrative role, receptionists are being increasingly relied on to fulfil clinically related tasks. Here we will produce guidance for receptionists and recommendations for how the processes they are involved in might be improved.

The application of rules, guidelines, regulations and protocols for these key tasks will never fully eradicate the imperfect and contingent nature of everyday work practices. Therefore, practices will be encouraged to customise or adapt our recommendations to meet the specific needs of their organisation and its patients. As such, they will also raise awareness among colleagues and policymakers of the responsibilities placed on receptionists in modern primary care.

## Ethics and dissemination

### Ethics

The protocol has been independently reviewed by external reviewers at the Health Foundation.^100^

### Dissemination

Our work will be disseminated using conferences, workshops, trade journals, electronic media and through a series of publications in the peer reviewed literature. The conferences will be carefully selected and used to present our work in terms of the results and the lessons learnt for future service improvement. We will arrange a series of workshops inviting stakeholders from across the primary care community to discuss our findings and the content and implementation of our recommendations. We will further raise awareness of our work among primary care staff using trade journals such as Practice Manager and electronic media such as Pulse. We will use a dedicated web page hosted by the University to serve as a central point of contact and as a repository of our findings. Finally, the study will produce a minimum of three articles for the international scientific literature and we hope will provide the basis for a comparison with similar roles elsewhere. The integration of rigorous research with state of the art tools of service improvement will itself draw attention to the findings and contribute to the methodology of improvement techniques.
